# Estimation on local transmission of malaria by serological approach under low transmission setting in Myanmar

**DOI:** 10.1186/s12936-017-2170-8

**Published:** 2018-01-05

**Authors:** Myat Htut Nyunt, Than Naing Soe, Thinzar Shein, Ni Ni Zaw, Soe Soe Han, Fauzi Muh, Seong-Kyun Lee, Jin-Hee Han, Ji-Hoon Park, Kwon-Soo Ha, Won Sun Park, Seok-Ho Hong, Myat Phone Kyaw, Eun-Taek Han

**Affiliations:** 1 Department of Medical Environmental Biology and Tropical Medicine, School of Medicine, Kangwon National University, Chuncheon, Republic of Korea; 2 Department of Medical Research, Yangon, Myanmar; 3 Department of Public Health, Nay Pyi Taw, Myanmar; 4 Department of Cellular and Molecular Biology, School of Medicine, Kangwon National University, Chuncheon, Gangwon-do Republic of Korea; 5 Department of Physiology, School of Medicine, Kangwon National University, Chuncheon, Gangwon-do Republic of Korea; 6 Department of Internal Medicine, School of Medicine, Kangwon national University, Chuncheon, Gangwon-do Republic of Korea

**Keywords:** Malaria, Serological surveillance, Asymptomatic cases, Myanmar

## Abstract

**Background:**

As the prevalence of the malaria has been decreasing in many endemic countries including Myanmar, malaria elimination in Greater Mekong Region was targeted not later than 2030. The relevance of molecular and serological tools to identify residual transmission remains to be established in this setting.

**Methods:**

One-year cohort study was conducted and sera samples were collected in every 3 months with active and passive case detection for clinical malaria episodes by RDT, microscopy and molecular method. The sera were used to detect the malaria antibody against PfMSP1-19, PvAMA1, PvDBPII and PvMSP1-19 by protein microarray.

**Results:**

Among the recruited 1182 participants, there was no RDT positive case for malaria infection although two vivax infections were detected by microscopy in initial collection. Molecular methods detected the asymptomatic cases of 28/1182 (2.37%) in first, 5/894 (0.42%) in second, 12/944 (1.02%) in third, 6/889 (0.51%) in fourth collection, respectively. Seropositivity rates against the PfMSP1-19, PvMSP1-19, PvAMA1 and PvDBPII were 73/270 (27.0%), 85/270 (31.5%), 65/270 (24.1%) and 160/270 (59.3%), respectively. PfMSP1-19 and PvMSP1-19 showed high and stable antigenicity in acute and subacute samples but declining in 1-year history samples. No cross reactivity of PfMSP1-19 and PvMSP1-19 between the two species and higher seropositivity among the asymptomatic carriers were observed. Mapping data indicated serological surveillance can detect the geographical pattern of malaria infection under low transmission setting.

**Conclusions:**

These findings support that PfMSP1-19 and PvMSP1-19 are suggested for serosurveillance of the malaria especially in low transmission setting for further necessary actions have to be carried out to eliminate the malaria.

## Background

In the *era* of (pre) elimination of malaria, surveillance is vital to estimate the local transmission of malaria [1]. Under the (pre) elimination phase, only sporadic cases are common and very few cases of fevers are due to malaria. Imported cases may represent the majority of the disease burden [2]. Currently, parasitological based cases detection with active and passive approach is widely used to detect and treat the clinical cases in the community [3]. Although the World Health Organization (WHO) recommends the parasitological diagnosis using rapid diagnostic tests (RDTs) or peripheral blood smear examination by microscopy, the detection limits of the above methods are not satisfactory to detect the low level parasitaemia [4]. Although molecular based detection methods are developed, these methods cannot be used widely in most of the field conditions because of the technical constraints and cost. Moreover, as the parasite densities fluctuate over time, leading to temporal variation in the detectability of infections and cross sectional analysis of the parasitological based detection method cannot estimate the local transmission of malaria correctly. To overcome it, one of the promising approaches is serological surveillance.

Unlike many other infectious diseases, malaria antibodies against the parasite antigens are widely diverted and some may last for long duration [5, 6]. As the antibody status may not reflect the acute malaria infection, it is not a suitable tool for diagnosis [7]. However, malaria antibody shows the local transmission profile indicating the indigenous malaria cases in the community [8]. Moreover, malaria antibody level was higher in older age assuming the cumulative exposure of the antigens. Significant lower level of malaria antibody was observed in high attitude reflecting the local transmission. In hyper endemic areas, local transmission of malaria can be assessed by malariometric survey and antigen assays as malaria cases are detected throughout the year. Under low endemic setting, malaria antibody is suggested for estimation of hot-spot by estimation on the geographical pattern of malaria transmission [9]. However, malaria antibody shows the complexity in nature, resulting from species, stage and strain specific antigen diversity [10–12]. Moreover, age specific antibody response was reported that may reflect the cumulative exposure or behaviour-related differences in exposure [13].

Although malaria serological analysis was reported, there are no validated serological marker(s), no standardized high accuracy detection platform and no interpretation approach [14]. Many studies were focusing on antibodies against the exo-erythrocytic and erythrocytic stages of the parasite. In this study, seroepidemiology and serokinetics of the PfMSP1-19, PvMSP1-19, PvDBPII and PvAMA1 were assessed to evaluate the usefulness as the serological markers for local transmission of malaria.

## Methods

### Study design and study population

One-year longitudinal follow-up sample collections were done in Shwegyin Township (22° 20′ 0″ N, 95° 56′ 0″ E), one of the Tier I areas of Myanmar Artemisinin Resistance Containment (MARC) areas in 2015. Because of the nearby gold mines, many migrant workers were working in these areas and malaria was notified as one of the leading diseases in Shwegyin and evidence on drug resistant-malaria was documented [15]. It has been selected as one of the township for elimination programme initiated in Myanmar.

### Sample size determination

Required sample size has been calculated by PASS sample size software (version 13, NCSS, USA). As this study was carried out as a longitudinal observational cohort, group sequential Log-rank test has been applied assuming 90% power to detect the hazard rate 0.76 when the proportions sero-conversion in each group were 0.3 and 0.4 at a significant level (alpha) of 0.05 using a two-sided Log-rank test for the four sequential tests in each active sample collection. The required minimal sample size is 884. Loss to follow-up had to be assumed 20% in each time and the sample size had to be 1060 in minimum.

### Sample collection procedures

The participants in this study were recruited by randomized cluster sampling in Shwegyin Township. Inclusion criteria included minimum age of 5 year, both sex and local residents in the study area for more than 3 years. As the study aimed to conduct a follow-up longitudinal study up to 1 year, the migrant or mobile populations were excluded. The person who currently showed the severe sign and symptoms of malaria was also not included in this study.

At each visit, 1 mL of the whole blood was collected from the fore arm of the participants under aseptic condition. The sera was extracted from the blood and kept at − 80 °C until analysis. The remaining whole blood was used for malaria parasite detection as described below.

### Sampling procedure

Randomized cluster sampling method was used in this study. According to the distribution of the local health centers, two out of the four centers were randomly selected. Then, all villages were listed, from which seven villages were randomly selected. Among them, sampling interval was calculated to get the required samples in the villages i.e., at least 151 per village. Household visit or public meeting places were used to collect the samples based on the convenience of the participants. Active case detection was done in every 3 months until 1 year and passive case detection was carried out by local health personnel.

### Malaria naïve samples

For negative control, healthy sera were collected from the people of the Republic of Korea who had no exposure to malaria. A total of 96 healthy malaria-naïve individuals were collected and they were 6–13 years old with mean age of 10 years.

### Detection on asymptomatic infection

Rapid diagnostic test (RDT) (PfHRPII and Pv specific pLDH based assay, SDFK80, Standard Diagnostics, Republic of Korea), microscopy and molecular method using nested PCR amplifying the small subunit ribosomal RNA, were used for detection of asymptomatic malaria infection in all samples collected in every 3 months as described details previously [16, 17].

All malaria infections were treated according to the National Malaria Treatment Guideline of Myanmar. Briefly, artemisinin-based combinational therapy (ACT) with primaquine stat dose for falciparum infection, chloroquine followed by 14 days course of primaquine in vivax infection and chloroquine for malaria infection.

### Serokinetic screening by protein microarray

**Fig. 1:**
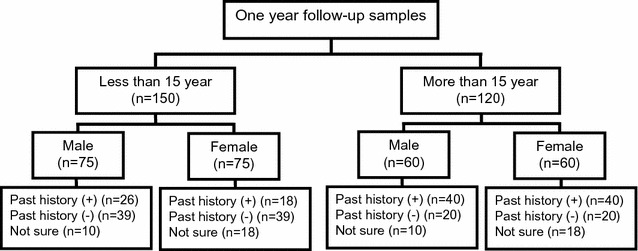
Selection of the sera for serological assessment among the participants

Among the collected samples, 685 (57.95%) attended all four visits. Among them, 270 samples were selected after excluding of the possible confounding factors, i.e., two cohorts in which composed of less than 15 years-old and more than 15 years-old samples in which equal distribution of male and female, and history of malaria (Fig. 1).

The procedures for protein array were described previously [18]. Briefly, amine coated slides were prepared. For microarray screening, 1 µL of optimized concentrations (25 ng/µL for PvMSP1-19, 12.5 ng/µL for PfMSP1-19, 100 ng/µL for PvDBPII and PvAMA1) was spotted into each wells of the arrays and incubated for 2 h at 37 °C. Then the slides were washed with PBS-Tween (0.1%) for 10 min followed by distilled water washing for 5 min. The array slides were then blocked with 5% BSA in PBS-T (PBS with 0.1% Tween) for 1 h at 37 °C. After washing again, the slides were probed with plasma collected from the patients and healthy control individual (1:25 dilution). Alexa Fluor 546-conjugated goat anti-human IgG (10 ng/µL, Invitrogen, Carlsbad, CA, USA) in PBS-T was used as the detection antibody and the signals were detected in a fluorescence scanner (InnoScan 300, Innopsys, Carbonne, France). The Mapix software was used for data acquisition and analysis. All samples were duplicated in the same slides. Mean values of two mean fluorescence intensity (MFI) were calculated.

### Cross reaction analysis

To assess the cross reactivity of the recombinant antigens between falciparum and vivax, protein microarray using pooled clinical falciparum, pooled clinical vivax and negative control pooled samples, was used in the same array slide with duplicated spots followed by the same procedures described above [19].

### Data analysis

Data was checked and analysed by using Microsoft excel and IBM SPSS Statistics (version 23, International Business Machines Corp., Armonk, NY, USA). Pearson’s Chi squared test was used to determine association with a *p* value of < 0.05 accepted as significant. Logistic regression was calculated using the selected independent variables to estimate the outcome variables. Mann–Whitney test was used to for nonparametric analysis on non-normal distribution. For microarray data, mean fluorescence intensity (MFI) data were normalized and transformed by *vsn* method with *asinh* (hyperbolic *arc sine*) using R-program [20]. The cut-off value was defined as two standard variation (SD)s above the transformed MFI of malaria naïve individual. Mapping for surveillance of the malaria cases by molecular method, PCR comparing with serological response against PvDBPII, PvAMA1, PvMSP1-19 and PfMSP1-19 was carried out. The map was generated as described previously using ARC GIS and SaTScan software [21]. Circular window was used to scan systematically adjusting with the percent positive among the participants in each villages. As the village level output is not provided by this software, final map was generated by the Photoshop CS3 software. In the map, the relative size of the circular windows for seropositive in each of the antigens in the study areas focusing on the geographical distribution of the malaria parasitaemia (by molecular) contracted with malaria serology positive (by protein microarray) were generated. The data include all four active sample collections in accordance with the sample size calculation by Sequential Rank Test.

### Ethical approval and consent to participate

The study was conducted only after receiving the ethical approval from Institutional Ethical Committee of the Department of Medical Research, Myanmar (Approval number 49/Ethics-2014). It was also registered with ClinicalTrial.gov (Identifier: NCT02708199). Written informed consents were taken in all of the participants. Participation in this study was entirely voluntary. All detected malaria cases were treated according to the national anti-malarial treatment guideline in Myanmar. The personal information collected in this study was kept confidential.

## Results

### Demographic characteristics of the study population

In this study, a total of 1182 local residents were recruited to study. Passive case detection was carried out by local health authorities and local malaria volunteers to detect the clinical malaria episodes. Active case detection and field blood sample collections were conducted one in every 3 months until 1 year. A total of 685 (58.0%) attended all four visits (Table 1).

**Table 1:** Basic demographic characteristics of the study population

Total recruited participants	1182
Age in year (median, IQR)	30 (18–45)
Sex (M:F)	4:5
History of malaria (n, %)	549 (46.4)
History of malaria within 1 year (n, %)	71 (6.0)
History of malaria within 1–3 years (n, %)	215 (18.2)
History of malaria more than 3 years (n, %)	263 (22.2)
Visit 1 (V1) collection (n = 1182, 100.0%)	
All asymptomatic cases^a^	30 (2.5%)
*P. vivax*	24
*P. falciparum*	4
*P. malariae*	2
Visit 2 (V2) collection (n = 894, 75.6%)	
Asymptomatic cases	6 (0.7%)
*P. vivax*	6 (0.7%)
Visit 3 (V3) collection (n = 944, 79.9%)	
Asymptomatic cases	13 (2.5%)
*P. vivax*	13 (2.5%)
Visit 4 (V4) collection (n = 889, 75.2%)	
Asymptomatic cases	8 (0.9%)
*P. vivax*	8 (0.9%)

^a^Asymptomatic cases were detected by nested PCR

### Detection of asymptomatic infection

All collected samples were checked for asymptomatic parasitaemia by RDT, microscopy and nested PCR. Although there was no RDT positive case, two cases in visit 1 were detected as *P. vivax* infections (parasite densities of 580 and 1200 parasites per µL, respectively). Among the participants, 39 showed asymptomatic infection by PCR and 12 were detected vivax infection in more than one visit (Table 1).

### Initial screening of selected candidate antigens for malaria antibody kinetic analysis

The purified recombinant target antigens were used to screen the four visits collected plasma samples of 270 participants by protein microarray. A total of 1080 plasma samples (1:25 dilution) and 98 healthy sera were included to analyze. Among the candidate serological markers, PvDBPII showed the highest seropositivity: 160/270 (59.3%) in at least one visit of sample collection, followed by PvMSP1-19 antigen 85/270 (31.5%), PfMSP1-19 antigen 73/270 (27.0%) and PvAMA1 antigen 65/270 (24.1%), respectively.

**Fig. 2:**
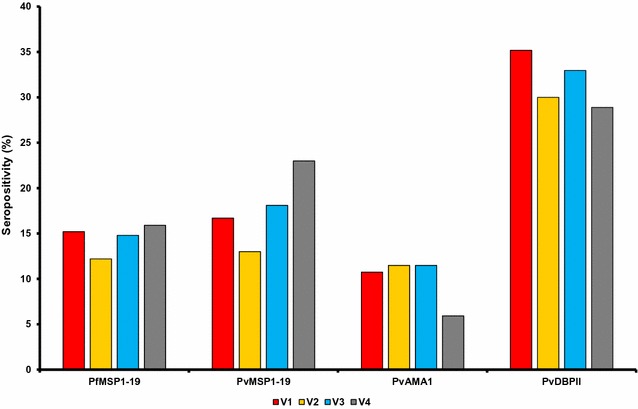
Seropositivity of the four different antigens among four visit (V1–V4)

Similarly, overall seropositivity in four times visits (V1-V4) data showed the highest rate in PvDBPII (31.7%), followed by PvMSP1-19 (17.7%), PfMSP1-19 (14.5%) and PvAMA1 (9.9%), respectively. PvDBPII showed the highest seropositivity and stable level among the study population in all four visits. Similarly, PfMSP1-19 showed the stable antigenicity among the population. However, PvMSP1-19 seropositivity showed the highest antigenicity in V4. Interestingly, PvAMA1 seropositivity was lowest in V4 (Fig. 2).

When the age group (< 15 and > 15 year) was considered as a factor that may contribute to the seropositivity of the candidate antigens, only PfMSP1-19 and PvDBPII showed higher seropositivity in old age group (*p* = 0.000 and *p* = 0.004). However, there was no association between the seropositivity of the antigens and sex of the participants (Table 2).

**Table 2:** Seropositivity against four different antigens in study population

Category	PfMSP1-19		PvMSP1-9		PvAMA1		PvDBPII	
	n (%)	*p*	n (%)	*p*	n (%)	*p*	n (%)	*p*
Age (year)								
< 15	18 (30.0)	0.000	45 (12.0)	0.599	43 (28.7)	0.062	77 (51.3)	0.004
> 15	55 (33.3)		40 (45.8)		22 (18.3)		83 (69.2)	
Sex								
Male	40 (29.6)	0.411	36 (26.7)	0.116	34 (25.2)	0.776	73 (54.1)	0.107
Female	33 (24.4)		49 (36.3)		31 (23.0)		87 (64.4)	
History of malaria within 3 years								
Yes	40 (32.2)	0.025	46 (37.1)	0.098	27 (21.8)	0.710	75 (60.5)	0.171
No	31 (26.3)		29 (24.6)		31 (26.3)		73 (61.9)	
Not sure	2 (7.1)		10 (35.7)		7 (25.0)		12 (42.9)	

Similarly, there was no association between the previous history of malaria and antibody seropositive rate except in PfMSP1-19 that showed the significant association (*p* = 0.0025). All antigens showed high seropositivity among the asymptomatic infection. PfMSP1-19 showed 3/4 (75.0%) seropositivity among asymptomatic falciparum infection while PvMSP1-19 showed 39/51 (76.5%) seropositivity among the asymptomatic vivax infection. Similarly, PvDBPII and PvAMA1 showed 37/51 (72.5%) and 38/51 (74.5%), respectively among the asymptomatic vivax infection (Table 2).

Among 270 samples, only 28/270 (10.4%) showed co-seropositivity against PvMSP1-19 and PfMSP1-19 antigens. On the other hand, PvMSP1-19 seropositivity alone accounted for 57/270 (21.1%) and PfMSP1-19 seropositivity alone for 45/270 (16.7%). When PfMSP1-19 and PvDBPII seropositivity rates were compared, 49/270 (18.2%) showed co-seropositivity. While PfMSP1-19 seropositivity alone accounted for 24/270 (8.9%), PvDBPII seropositivity alone was 111/270 (41.1%). Similarly, seropositivity rates of PfMSP1-19 and PvAMA1 were compared and 13/270 (4.8%) were seropositive against both antigens. Similar seropositivity rate against the PfMSP1-19 alone (60/270, 22.2%) and that against PvAMA1 alone (52/270, 19.3%) was observed.

Among the three vivax antigens, seropositivity rates of PvMSP1-19 and PvDBPII were compared as these two candidates showed the highest seropositivity rate. Only 50/270 (18.5%) were seropositive against both antigens. While 110/270 (40.7%) were seropositive against PvDBPII only, 35/270 (13.0%) of the samples showed seropositivity against PvMSP1-19 (*p* = 0.0034).

**Fig. 3:**
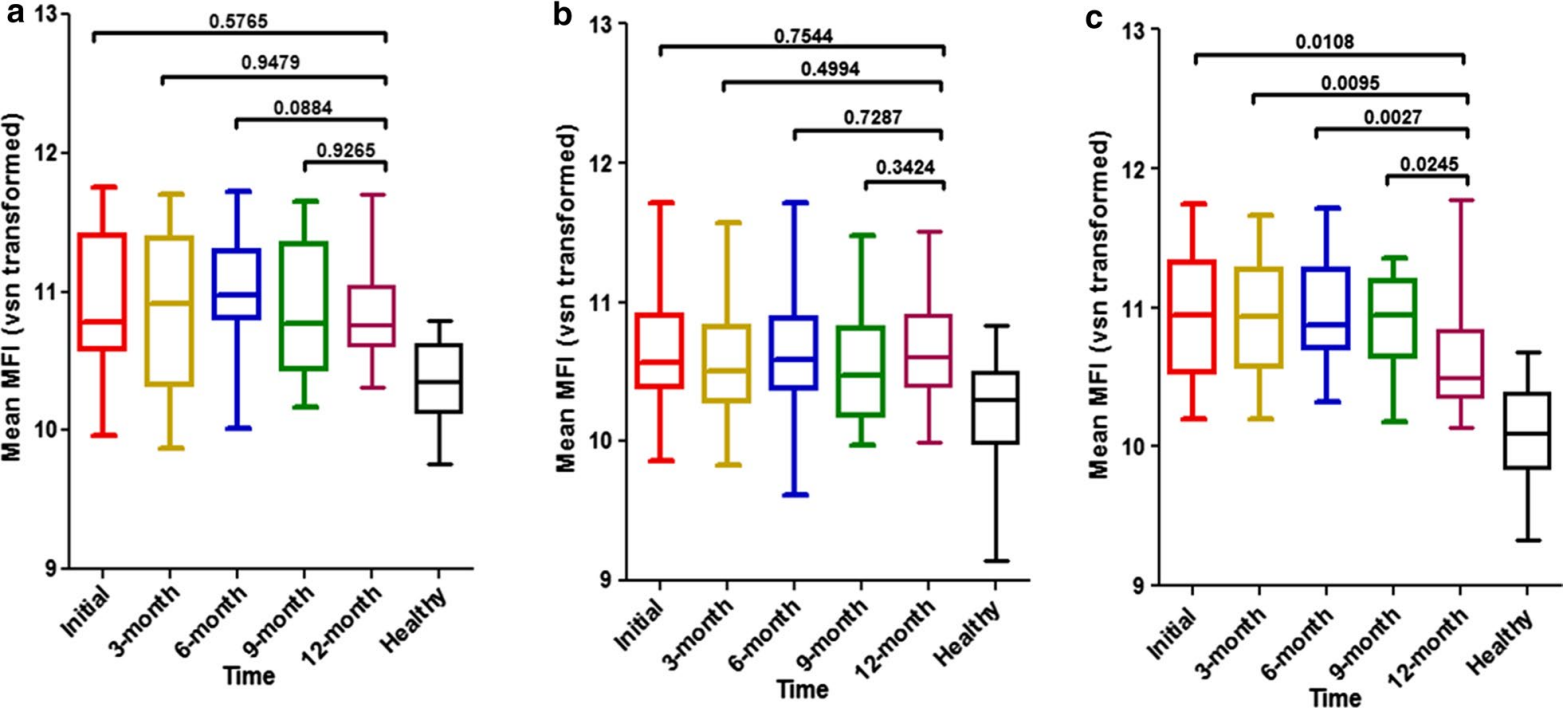
Serokinetic of the vivax antigens among the asymptomatic vivax infected cases

Serological response among the four vivax antigens among vivax subclinical cases indicated that all antibody level were stable up to 1 year after infection except in PvMSP1-19 which showed significant reduction of mean fluorescence intensity in 9 months after treatment of infection (Fig. 3).

### Geographical patterns in seropositivity and parasitaemia

**Fig. 4:**
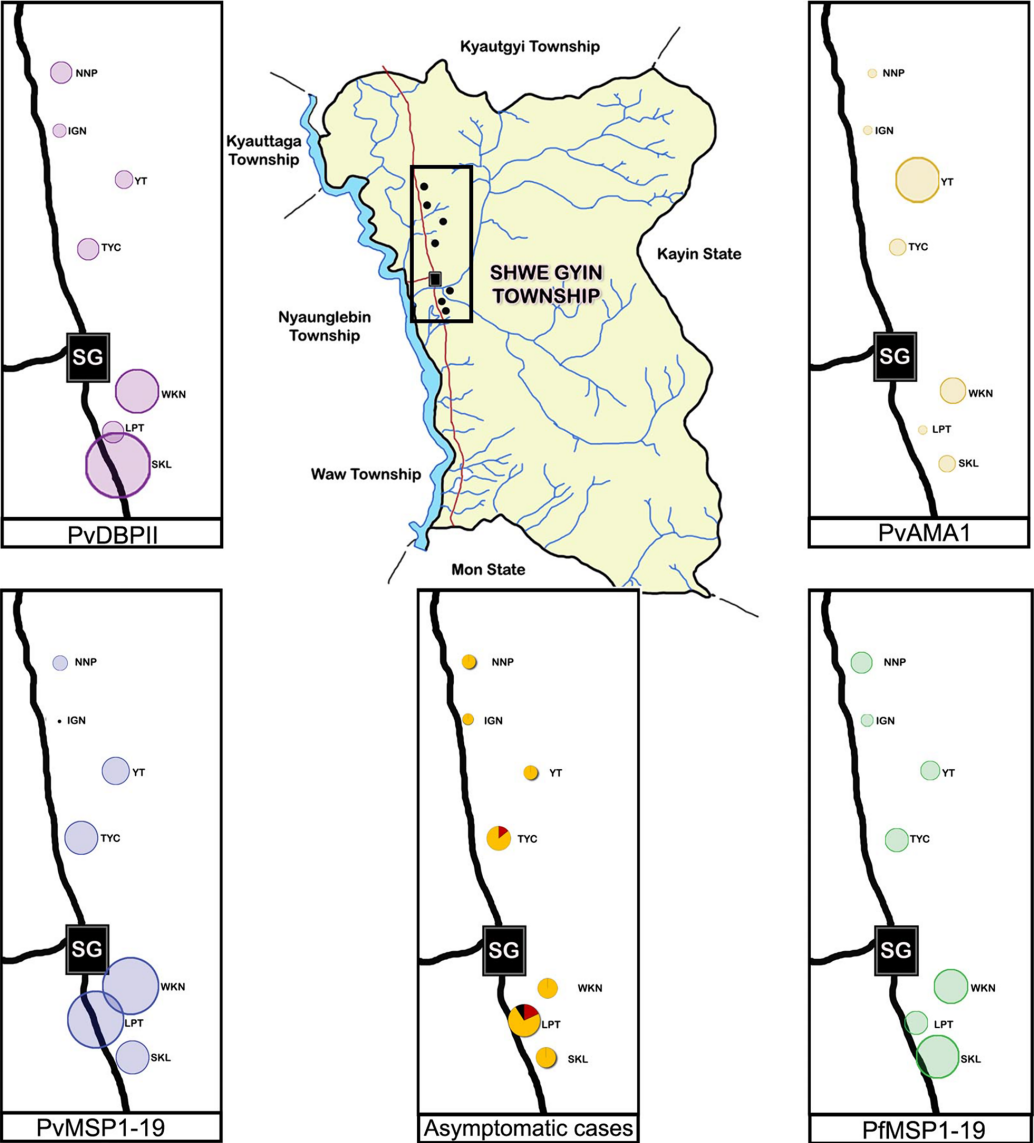
Mapping of the seroprevalence of the four different targets and molecular confirmed subclinical cases. The size of the circle represents the relative percent of the positive cases

When mapping was done to detect the malaria geographical patterns of malaria in seropositivity compared with parasitaemia in the samples collected in active case detection by PCR, the PvMSP1-19 showed the similar pattern of the local transmitted areas with that by molecular method. PvAMA1 and PvDBPII showed the high seropositivity in the villages at which very few vivax asymptomatic cases were identified by PCR, suggesting the long-lasting antibody response against these antigens (Fig. 4).

## Discussion

Malaria serology is a cornerstone in estimation on local transmission of malaria. Although there are many studies on malaria serology [22–24], there is no standardized method for recombinant antigens production, no standardized approach for detection of antibody status and no validated antigen for all transmission setting.

In this study, the recombinant antigens; PfMSP1-19, PvMSP1-19, PvAMA1 and PvDBPII were screened in 1080 samples collected from 270 participants in every 3 months for 1 year. Among them, the highest seropositivity was observed in PvDBPII, followed by PvMSP1-19, PfMSP1-19 and PvAMA1.

PvDBPII is the most interesting vaccine candidate and widely studied for vaccine [25]. However, antibody responses showed the relationship with the heterogeneity of the parasite population [26]. The humoral immune antibody response against PvDBPII is high and stable in the population regardless of the history of past infection in the present study. Serological markers should not be the vaccine candidate to exclude the humoral immune response induced by vaccine [27]. Moreover, a higher seropositivity rate of PvDBPII was observed in older age group indicating the long-lasting nature of the antibody [5]. Due to the long-lasting antibody status and potential vaccine candidate, PvDBPII is not suitable for serological marker to estimate the local transmission of malaria.

The humoral immune response against PvMSP1-19 also showed the stable kinetic among all four sample collections in this study. The presence of the PfMSP1-19 antibody can prevent against the parasitaemia and malaria related febrile illness [28]. Not only the AMA1 but also the MSP1-19 antibody were found to have protective activity against symptomatic malaria [29]. However, vaccine-induced antibody was not protective against the vaccine-dissimilar strains due to the strain specific antibody response [30]. Moreover, high and stable seropositivity was also noted in all four visit collections leading to consider a potential vaccine candidate. High polymorphism in PvAMA1 gene influences the antigen specific response that limit the usefulness of the PvAMA1 as vaccine or serological marker [31].

One study conducted in Uganda [32] found that MSP1-19 seroprevalence and parasite prevalence were decreased in high attitude hilly region, indicating the usefulness as a serological tool to estimate local transmission of malaria. In this study, both falciparum and vivax MSP1-19 antibodies were found to be stable. The presence of the antibody may prevent against the clinical or even asymptomatic infection. Moreover, antibody against MSP1-19 was observed in no longer than 9 months after infection indicating its usefulness as a serological marker to track the local transmission of malaria under low transmission setting.

Moreover, there was no cross reactivity of MSP1-19 antibody among the all four common *Plasmodium* species [33]. It was found that very low or no cross reactivity of MSP1-19 antibody to falciparum and vivax among the two natural infections, reflecting the low amino acid identity (40.2%) between the two species. Discrimination between the two species; falciparum and vivax is an important issue in the area previously or currently occupied by both species.

Interestingly, there was no malaria case among local resident populations by passive case detection while molecular method can detect the subclinical infection of 2.5, 0.7, 2.5 and 0.9% of the samples collected on visit 1–4. Most of them were vivax infection as a usual finding of the (pre)elimination area where previously occupied by both falciparum and vivax malaria. However, a limited number of the PCR confirmed asymptomatic cases were not allowed to analyze the correlation of the seropositivity rate between asymptomatic cases and non-infected samples.

Moreover, MSP1-19 antibody responses were similar geographical distribution to the parasitological based detection using PCR. Both falciparum and vivax MSP1-19 antibodies were found to be stable up to 9 months after infection, then decreased significantly indicating the promising candidates to be used to assess local transmission of malaria under low transmission setting.

One limitation of this study is that the strain specific antibody responses in the community against the strain specific antigen(s) were not determined. This may affect the overall seroprevalence of the antigens. Moreover, this study was conducted as a longitudinal observational study under low transmission setting for only 1 year period. To validate the evidence, a multisite cross sectional study including control site should be conducted in a different endemic setting.

## Conclusions

In summary, estimation on local transmission of malaria is a fundamental information for priority assessment, strategy making, planning, implementation and evaluation of the interventions in all malarious areas. Routine methods based on phenotypic and genotypic characteristics are not sensitive enough to estimate the local transmission of malaria in the community. Serological surveillance using the recombinant PfMSP1-19 and PvMSP1-19 may provide an alternate tool for estimation on local transmission of malaria under low transmission settings.
